# Quantitative 3D measurements of tibial plateau fractures

**DOI:** 10.1038/s41598-019-50887-6

**Published:** 2019-10-07

**Authors:** N. Assink, J. Kraeima, C. H. Slump, K. ten Duis, J. P. P. M. de Vries, A. M. L. Meesters, P. van Ooijen, M. J. H. Witjes, F. F. A. IJpma

**Affiliations:** 1 Department of Technical Medicine & MIRA Institute for Biomedical Engineering, University of Twente, Drienerlolaan 5, 7522 NB Enschede, The Netherlands; 2 3D Lab/Department of Oral and Maxillofacial Surgery, University of Groningen, University Medical Center Groningen, Hanzeplein 1, 9713 GZ Groningen, The Netherlands; 3 Department of Trauma Surgery, University of Groningen, University Medical Center Groningen, Hanzeplein 1, 9713 GZ Groningen, The Netherlands; 4 Department of Surgery, University of Groningen, University Medical Center Groningen, Hanzeplein 1, 9713 GZ Groningen, The Netherlands; 5 3D Lab/Department of Radiology, University of Groningen, University Medical Center Groningen, Hanzeplein 1, 9713 GZ Groningen, The Netherlands

**Keywords:** Fracture repair, Orthopaedics

## Abstract

Fracture gap and step-off measurements on 2DCT-slices probably underestimate the complex multi-directional features of tibial plateau fractures. Our aim was to develop a quantitative 3D-CT (Q3DCT) fracture analysis of these injuries. CT-based 3D models were created for 10 patients with a tibial plateau fracture. Several 3D measures (gap area, articular surface involvement, 3D displacement) were developed and tested. Gaps and step-offs were measured in 2D and 3D. All measurements were repeated by six observers and the reproducibility was determined by intra-class correlation coefficients. Q3DCT measurements demonstrated a median gap of 5.3 mm, step-off of 5.2 mm, gap area of 235 mm^2^, articular surface involvement of 33% and 3D displacement of 6.1 mm. The inter-rater reliability was higher in the Q3DCT than in the 2DCT measurements for both the gap (0.96 vs. 0.81) and step-off (0.63 vs. 0.32). Q3DCT measurements showed excellent reliability (ICC of 0.94 for gap area, 1 for articular surface involvement and 0.99 for 3D displacement). Q3DCT fracture analysis of tibial plateau fractures is feasible and shows excellent reliability. 3D measurements could be used together with the current classification systems to quantify the true extent of these complex multi-directional fractures in a standardized way.

## Introduction

Tibial plateau fractures represent 1–2% of all fractures in adults and are reported as one of the most challenging injuries of the knee^1^. Since the tibial plateau is among the most loadbearing areas in the body, any fractures affect knee alignment, stability and motion. Adequate treatment is crucial to minimize patient disability and other consequences (e.g., posttraumatic arthritis)^2^. Adequate classification and fracture assessment of tibial plateau injuries is essential in the choice of the right treatment strategy^3^. Currently, the Schatzker and AO/OTA are the most widely used and accepted classification systems^4–6^. Although these classifications represent the gross fracture patterns, they do not include detailed information about the severity of the dislocation, gap, and step-off^7,8^. Furthermore, it has been reported that these classification systems have their limitations, because of their moderate inter-rater reliability^3,9,10^.

The goal of any surgical treatment of the fractured tibial plateau is to restore the articular surface and provide a stable fixation, which allows immediate postoperative exercising^11^. A CT-scan provides information about the fracture anatomy, which helps in the physician’s choice between conservative or operative treatment. In clinical practice, the surgeon scrolls through the CT slices and tries to get a general impression of the fracture. Furthermore, the gaps and step-offs on the various slices can be measured. The physician’s assessment of the CT-scan, however, depends heavily on the way these are performed and which CT slice is selected for the measurements. Therefore, two-dimensional (2D) measurements of the CT scan can vary significantly between physicians. Moreover, it is difficult to quantify the true extent of the injuries from a few 2D CT slices, since articular incongruity frequently involves three-dimensional (3D) displacement (e.g, gaps and step-offs) in multiple planes^12^.

The importance of the three-dimensional aspect of tibial plateau fractures was recently acknowledged with the attempt to convert the most commonly used Schatzker classification to a new 3D classification system by the original author who posed this classification four decades ago^13^. In other complex fractures types has been demonstrated that the implementation of 3D technology for visualization, classification and surgical planning provides clinical benefits. The application of 3D technology in acetabular fracture surgery for instance improved education, classification, surgical planning and recently resulted in the development of patient-specific implants at our department^14–16^. Also, in the field of oral and maxillofacial surgery the use of 3D virtual planning and guided surgery is of substantial clinical value in order to perform complex jaw reconstructions within millimeters^17^. Recently, 3D statistical shape models of the tibia were introduced in this journal^18^. These models seem to be a promising tool for assessing anatomical variations and will be helpful in gaining a more patient-specific approach regarding fracture reduction techniques and implant fitting. In line with these developments, we present a quantitative 3D CT (Q3DCT) measurement tool for tibial plateau fractures. To our best knowledge, 3D measurement tools for these types of fractures are still lacking.

Quantitative 3DCT measurements have the potential advantage of representing the multidirectional (3D) aspect of fractures and they provide a uniform way of measuring the gap and step-off in these injuries^12,19–23^. The clinical applicability of these 3DCT measurements would be usage in addition to the current classification systems to assess initial and/or residual displacement, and they might eventually be related to patients reported outcome measures. The goal of this study was to develop a standardized 3D measurement tool to determine quantitatively the extent of the tibial plateau fractures.

## Methods

### Patients

Ten patients with a tibial plateau fracture were included in this study. All of them underwent surgery at the University Medical Center of Groningen (UMCG). They were included upon the availability of a pre-operative CT-scan of the injured knee with a slice thickness of 0.6 mm (pixel spacing: 0.44 × 0.44 mm). The CT data were used to develop a quantitative 3D measurement technique for tibial plateau fractures. All the fractures were graded according to the Schatzker and AO/OTA classification systems by using both preoperative plain radiographs and CT-scans. The institutional review board of the University Medical Center Groningen approved the study procedures and the research was performed in accordance with the relevant guidelines and regulations. Informed consent was obtained from all subjects.

### 3D Fracture models

**Figure 1:**
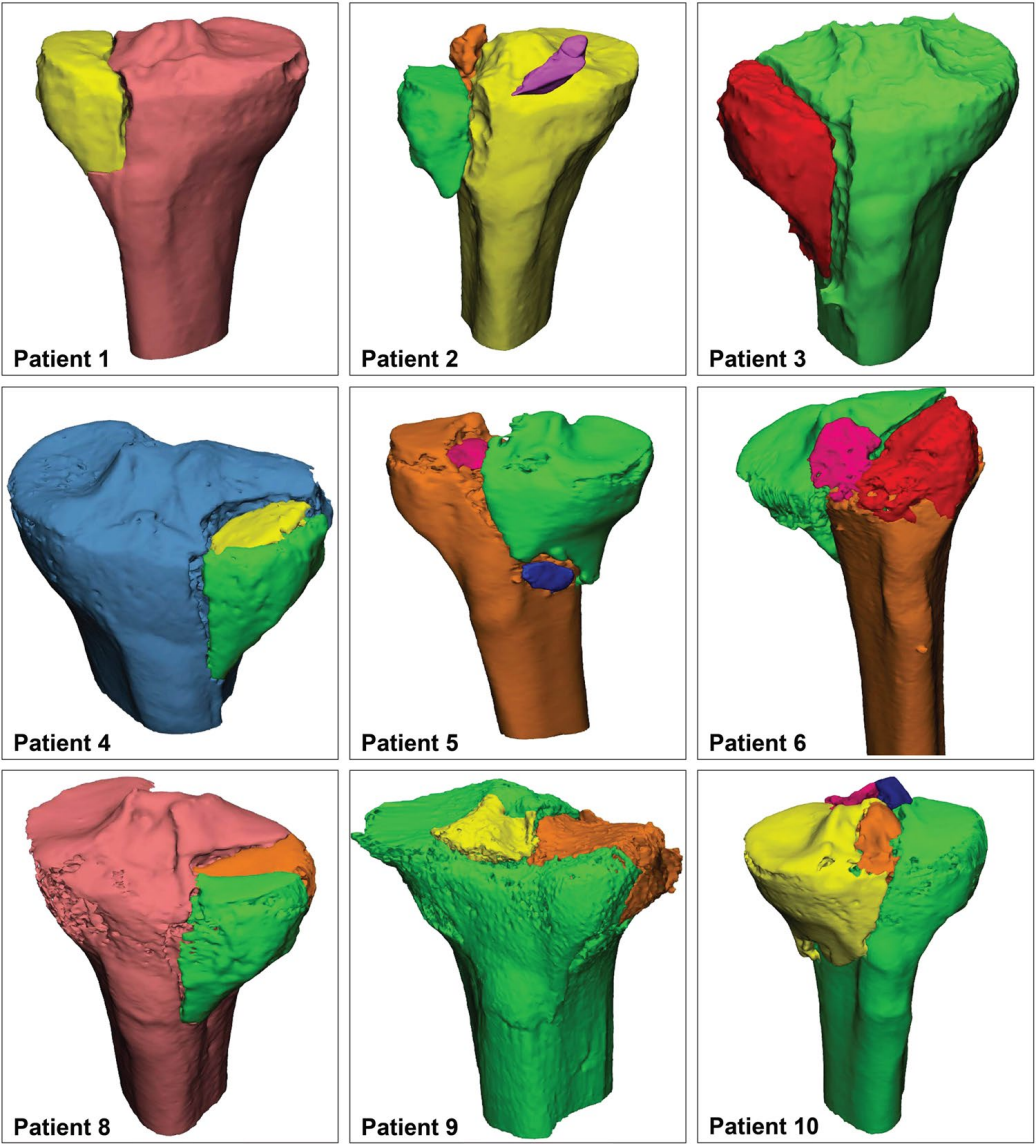
3D reconstructions of all the patients except for patient 7. This patient is used to illustrate the Q3DCT measurements in Figs 2–4.

**Figure 2:**
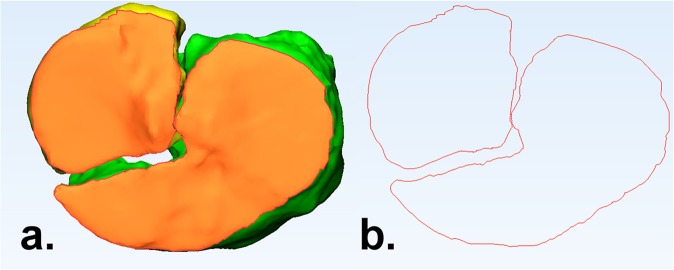
(**a**) The articular surface, in terms of the top axial view of the tibial plateau, is marked orange; (**b**) The contour of the articular surface, demonstrating the fracture pattern.

**Figure 3:**
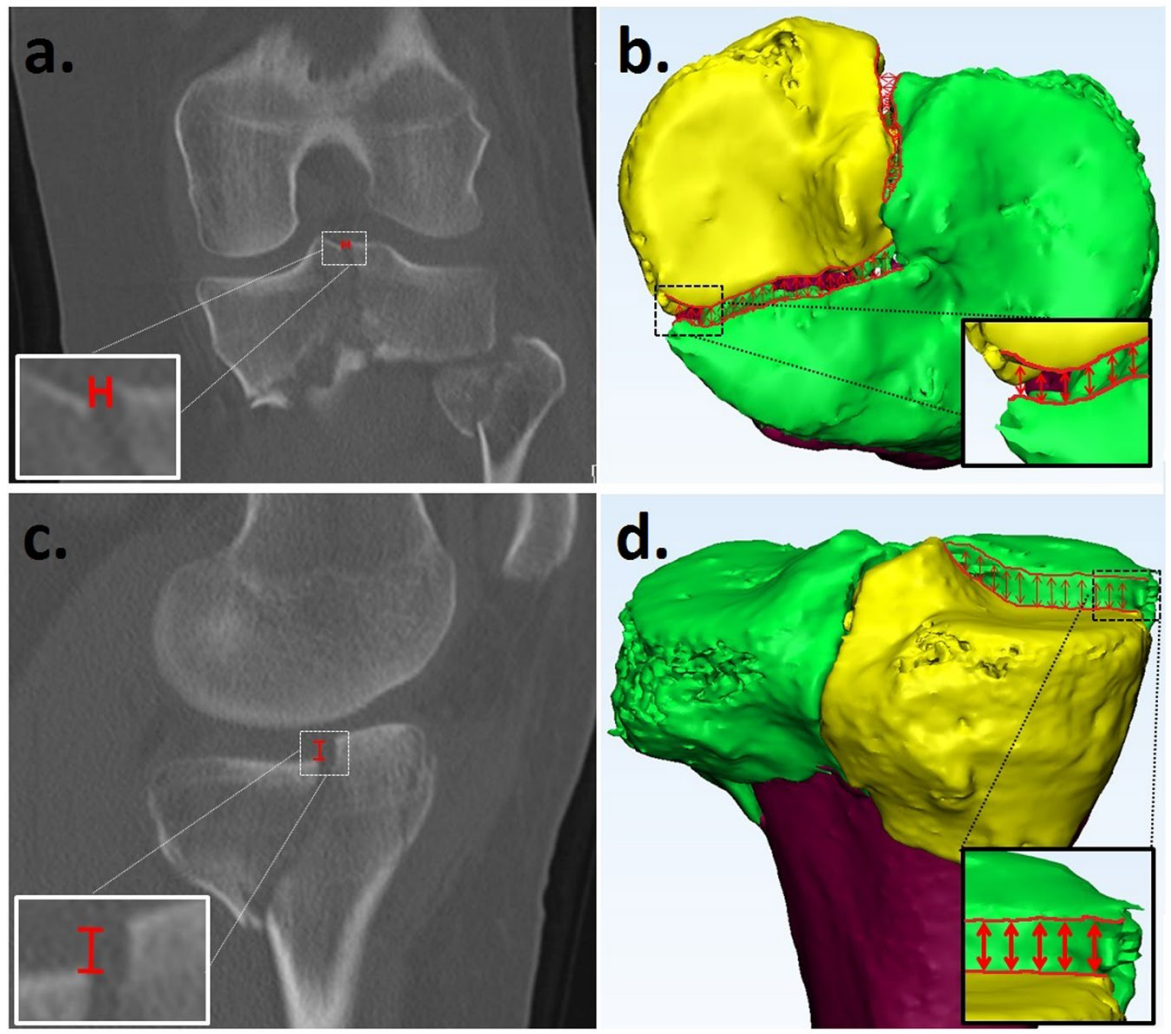
Gap measurement from a 2DCT slice (**a**) and a Q3DCT model (**b**). Step-off measurement on 2D (**c**) and Q3DCT. (**d**) The 2DCT slices had to be scrolled to find the maximum gap and step-off. The Q3DCT has the advantage that the gap and step-off could be measured between all points along the fracture lines within the same plane providing a maximum and a mean 3D value of both parameters.

**Figure 4:**
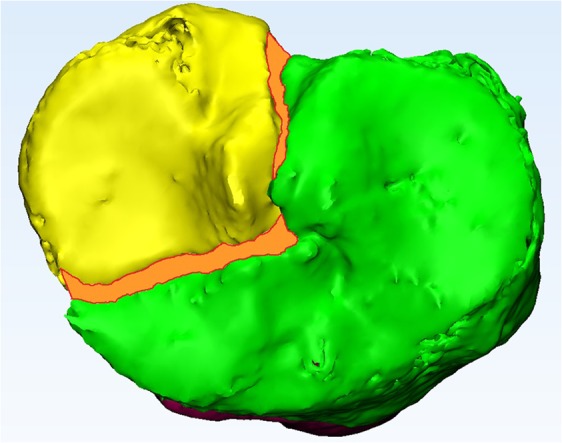
Measurement of the gap area (mm^2^), which is marked as the orange surface area between the fracture lines.

Mimics Medical software package (Version 19.0, Materialise, Leuven, Belgium) was used to create 3D models of all the injured knees. First, the CT data (DICOM files, Digital Imaging and Communications in Medicine) were imported. Secondly, a segmentation process was performed by using a preset bone threshold (Hounsfield Units ≥226) combined with region growing in order to separate the independent fragments. Subsequently the fragments were checked and if needed manually separated from adjacent fragments. Smoothing was applied (factor 0.4) and each fragment was assigned a different color. Subsequently, virtual anatomical reduction of the fragments was performed. The fragment was moved to its anatomical position and exactly fitted on a template of a healthy tibia. The accuracy of the reduction was checked and approved by two surgeons. Figure 1 represents the 3D models of nine of the ten included patients (the 3D model of patient 7 is presented in Figs 2–4).

### Quantitative 3D measurements

The 3D measurements were conducted by using both the 3-matic Medical (Version 11.0, Materialise, Leuven, Belgium) and Matlab (R2014B, Mathworks, Natick, Massachussetts, US) software. First of all, the edges of all the fracture fragments at the level of the articular surface were determined by using the 3-matic (Fig. 2). The “classic” gaps and step-offs were measured in Q3DCT. Furthermore, three additional 3D parameters were introduced, namely the gap area, articular surface involvement and 3D-displacement. The details of these measurements are clarified below.

#### Gap and step-off

The 3D articular gaps and step-offs were determined by calculating the differences in distance and height between the fracture lines of adjacent fracture fragments at the articular level (Fig. 3). In order to calculate the gap and step-off, the fracture lines were identified and exported from the 3-matic to the Matlab software. A gap was defined as a separation of fracture fragments *along* the articular surface. A step-off was characterized as a separation of fracture fragments *perpendicular* to the articular surface. Besides the maximum gap and step-off, the mean of the gaps and step-offs were determined between all the points along adjacent fracture lines in the 3D model.

Furthermore, the maximum gap and step-off in the coronal, sagittal and axial plane of the 2DCT slices were measured according to current practice in order to compare them with the 3DCT measurements. The gaps and step-offs were measured in the CT slices and the largest gap and step-off was used for the analysis (Fig. 3a,c).

#### Gap area

The gap area is defined as the total surface area of the gap between all fracture fragments. It was measured by calculating the surface area (mm^2^) between all the fragment fracture lines, which were projected in one plane at the articular level (Fig. 4).

#### Articular Surface involvement

Articular surface involvement represents to what extent the articular surface is damaged due to the fracture. It was determined by dividing the sum of all the articular surface areas of the displaced fracture fragments by the total joint surface of the tibial plateau according to formula 1.

1 $$Articular\,surface\,involvement=\frac{\sum Fragmen{t}_{i}}{Total\,surface}\times 100 \% $$

#### 3D displacement

**Figure 5:**
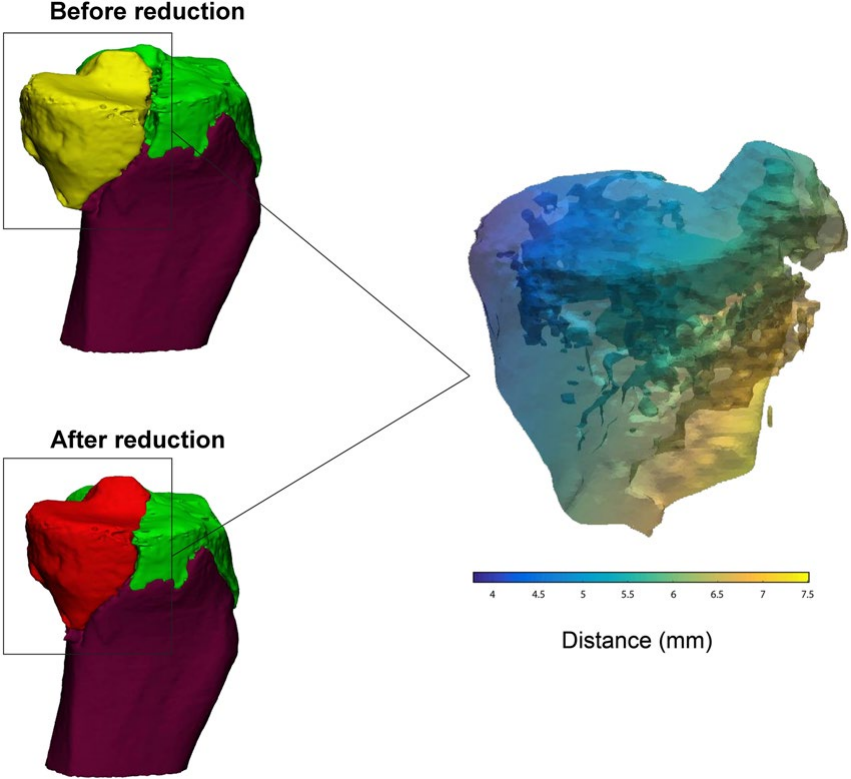
Measurement of the 3D displacement (mm). Left: fragment of the tibial plateau fracture before (yellow) and after (red) reduction. Right: distance map representing the difference in the fragment’s position before and after the reduction (3D displacement). The colour corresponds with the severety of the displacement, whereby yellow represents a relatively big displacement and blue represents a relatively small displacement.

The extent of dislocation of each fragment was determined by calculating its 3D displacement along the axis in three directions (x, y and z). In order to calculate the 3D displacement, the fracture has to be reduced in the 3D model. The 3D model of the fragment represents numerous surface points (vertices). The 3D displacement (in mm) was determined by the sum of the distances between every point before and after reduction according to formula 2. A 3D displacement is therefore determined for each fragment based on tens of thousands of single points. Furthermore the displacement of each part of the fragment can be presented as a distance map as shown in Fig. 5.

2 $$3D\,Displacement=\sum \sqrt{\Delta {x}_{i}^{2}+\Delta {y}_{i}^{2}+\Delta {z}_{i}^{2}}$$

### Statistical analysis

Statistical analysis was performed with SPSS (version 23, IBM, Chicago, IL, US). The Wilcoxon signed-rank test was used to evaluate differences between 2DCT and 3DCT measurements of the gaps and step-offs. A P-value of less than 0.05 was considered statistically significant. Furthermore, a Spearman’s rank-order correlation coefficient was used to assess whether there was a correlation between the maximum gap (measured in 2D and Q3DCT) and the gap area.

#### Reliability

To assess the reliability of the 3D measurements, six observers independently performed the Q3DCT measurement on all the cases. Furthermore, six observers measured the gap and step-off on 2DCT slices from all the cases. The Cohen’s Kappa was calculated to determine the agreement between the observers regarding slice selection and the maximum gap and step-off measurement from the 2DCT images.

The inter-rater reliability (IRR) was calculated by using intraclass correlation coefficients (ICC). We used 2-way mixed, single measurements and absolute agreement. Interpretation of the ICC values was performed according to the guidelines proposed by Cicchetti^24^ whereby IRR is considered to be poor when the ICC values are less than 0.40, fair when the values are between 0.40 and 0.59, good when the values are between 0.60 and 0.74 and excellent when the values are higher than 0.74.

In order to determine the reproducibility of the 3D-displacement, this measurement was repeated by one observer after a period of 4 weeks. The IRR was determined from the 3D-displacement calculated on the two separate occasions.

### Ethical approval

This study has been approved by the local medical ethical committee.

## Results

Ten patients (6 females, 4 males, mean age 43 (range 18–81)) with a tibial plateau fracture were included and 3D computer models of their fractures were created (Fig. 1). Five of them were treated with open reduction and plate fixation and five with percutaneous screw fixation only. The following fracture types were included; one patient with a Schatzker 1, five with a Schatzker 2, three with a Schatzker 4 and one with a Schatzker 5 fracture. According to the AO/OTA classification system, these injuries were classified as one 41-B1, eight 41-B3 and one 41-C1 fracture. The results of the 2D and Q3DCT measurements of the different types of fractures are presented in Table 1.

**Table 1:** Q3DCT and 2DCT measurements of the fracture characteristics in 10 patients who had suffered various types of tibial plateau fractures.

Patient	Classification		Q3DCT measurements									2DCT measurements	
No.	Schatzker	AO/OTA	Gap		Step-off		Gap area (mm^2^)	Articular surface involvement (%)	3D- displacement			Maximum Gap (mm)	Maximum Step-off (mm)
			Maximum (mm)	Mean (mm)	Maximum (mm)	Mean (mm)			Fragments (N)	Displaced fragments (N)	Displacement (mm)		
1.	1	41-B1	2.9	1.2	3.3	1.6	57	18.9	2	1	2.2	2.4	3.3
2.	2	41-B3	14.9	10.3	6.6	3.7	314	28.8	4	3	6.4; 47.2; 15.0;	11.2	7.8
3.	2	41-B3	8.5	5.6	3.7	1.7	254	12.7	2	1	5.0	7.4	3.1
4.	2	41-B3	3.9	1.8	6.1	4.8	28	10.5	3	2	4.3; 2.0;	3.3	5.1
5.	4	41-B3	18.3	14.2	3.7	1.8	510	78.0	4	3	11.3; 9.0; 8.1;	12.4	6
6.	4	41-B3	6.6	3.7	6.4	2.3	215	100.0	3	3	17.6; 10.8; 15.4;	7.3	0
7.	5	41-C1	4.4	2.2	5.0	2.6	298	37.3	2	1	5.7	3.9	4.6
8.	2	41-B3	2.8	2.0	5.9	1.7	18	15.3	3	2	2.7; 2.6;	2.8	2.9
9.	2	41-B3	2.7	1.0	6.6	3.2	77	39.7	3	2	3.6; 2.3	2.9	3.8
10.	4	41-B3	5.3	2.7	4.3	1.9	286	75.1	5	4	3.2; 6.7; 6.8; 5.6;	5.2	5.2
Median (IQR)	N/A	N/A	5.3 (4.3)	2.5 (3.3)	5.2 (2.3)	2.1 (1.3)	235 (233)	33 (50)	N/A	N/A	6.1 (7.1)	8.6 (7.8)	5.3 (1.7)

### Quantitative measurements

The maximum gaps and step-offs, as measured on the Q3DCTs, are presented in columns 4 and 6 of Table 1, respectively. Within the 10 patients, the measured maximum gaps showed a median value of 5.3 mm (IQR: 4.4), whereas the maximum step-offs had a median value of 5.2 mm (IQR: 2.3). The maximum gap and step-off measurements from the 2D CT slices resulted in higher median values of 8.6 mm (IQR: 7.8) and 5.3 mm (IQR: 1.7), respectively. No significant differences were found between both the maximum gap (P-value: 0.29) and step-off (P-value: 0.29) measured on the Q3DCTs and 2DCTs. The mean gaps and step-offs between all the points along the fracture lines were calculated for each case (Table 1; Columns 5 and 7) and showed values between 1–14 mm and 1.6–4.8 mm, respectively.

The gap area (column 8 of Table 1) was measured for each patient and demonstrated a median gap area of 235 mm^2^ (IQR: 233). The Spearman’s correlation coefficient between the maximum gap measured in 2D and the gap area was 0.94 (P < 0.001). The Spearman’s correlation coefficient between the maximum gap measured in Q3DCT and the gap area was 0.88 (P < 0.001). However, the maximum gap in some of the patients (cases 6, 7 and 10) was relatively small but the gap area was quite large due to a multitude of fracture lines with moderate gapping.

The articular surface involvement (column 9 of Table 1) could be determined for each patient and demonstrated a median value of 33% (IQR: 50). The three cases classified as Schatzker 4 (cases 5, 6 and 10) had the largest articular surface involvement with values of over 75%.

The 3D displacement (columns 10, 11 and 12 of Table 1) of each fracture fragment could also be determined for all the cases. The number of fragments in each injury and the degree of their displacement was established in the 3D models. These measurements showed the feasibility of determining the 3D displacement of individual fracture fragments. The mean 3D displacement of all the fragments in the various injuries was 6.1 mm (IQR: 7.1).

### Reliability

The inter-rater reliability (IRR) of the measured maximum gaps was found to be excellent with an ICC of 0.81 from 2DCTs. The reliability of the maximum gap measurements increased to 0.96 with Q3DCT (Table 2). The IRR of the step-offs was found to be poor from 2DCTs, with an ICC of 0.32 and good from Q3DCTs, with an ICC of 0.63. On measuring the mean gaps and step-offs in the 3D fracture model, the IRR improved considerably to 0.97 and 0.80, respectively. When measuring the maximum gaps and step-offs from the CT scans, the observers had selected different 2D slices. This led to a mean Cohen’s kappa of 0.12 and 0.09 for the slice selection agreements between the 2DCT gap and step-off measurements, respectively.

**Table 2:** Intra correlation coefficients (ICC) with their 95% conficence interval (CI) were determined for all the 2DCT and 3DCT gap and step-off measurements of all cases made by six observers.

Parameters	2DCT measurements	Q3DCT
Maximum Gap	0.81 (95% CI: 0.62–0.94)	0.96 (95% CI: 0.91–0.99)
Mean Gap	—	0.97 (95% CI:0.93–0.99)
Maximum Step-off	0.32 (95% CI: 0.1–0.67)	0.63 (95% CI: 0.35–0.87)
Mean Step-off	—	0.80 (95% CI:0.60–0.94)

**Figure 6:**
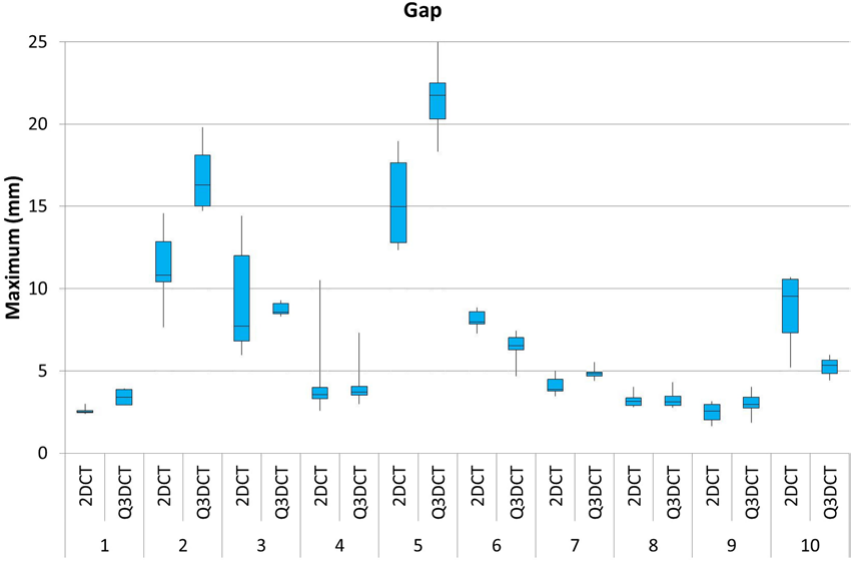
A boxplot showing the dispersion of the maximum gap measurements from 2DCT slices in comparison to our 3DCT measurements, performed by six different observers for all the patients.

**Figure 7:**
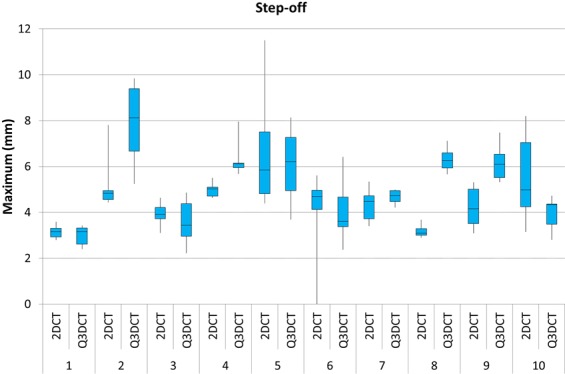
A boxplot showing the dispersion of the maximum step-off measurements from 2DCT slices compared to to our Q3DCT method, performed by six different observers for all patients.

Figures 6 and 7 depict the degree of dispersion between observers when performing the 2DCT and Q3DCT measurements of the maximum gap and step-off for each patient. There was more dispersion in the 2DCT measurements of the gap in 6 out of the 10 patients compared to the Q3DCT measurements. Regarding, the measurements of the step-off, the dispersion between 2DCT and Q3DCT was equally distributed.

There was excellent reliability between the different observers for the additionally introduced Q3DCT measurements. The gap area showed an ICC of 0.94 (95% CI: 0.85–0.98), the articular surface involvement had an ICC of 1 (95% CI: 0.99–1), and the 3D-displacement demonstrated an ICC of 0.99 (95% CI: 0.96–1).

## Discussion

This study presents a quantitative 3DCT measurement technique to determine the 3D fracture characteristics of tibial plateau fractures. This study demonstrates that accurate intra-articular gap and step-off measurements in 3D are feasible for tibial plateau fractures. Furthermore, three additional 3D measurement tools are presented, namely the gap area, articular surface involvement and 3D-displacement of individual fracture fragments, to gain a better three dimensional insight of the fracture.

Recently, Millar *et al*. extensively reviewed current classifications for tibial plateau fractures and they identified no less than 38 classification systems^3^. Moreover, most of these classification systems had moderate intra- and inter-observer reliability and did not provide quantitative information about the fracture patterns and morphology. They suggested the use of more sophisticated imaging modalities such as 3D CT to improve reliability estimates.

Q3DCT measurements have some potential advantages in comparison to 2DCT measurements. First of all, the inter-rater reliability of 3D measurements is higher than 2DCT measurements in the current analysis. The results of the 2DCT measurements depend heavily on the slice selection and the way gaps and step-offs are interpreted from different angles (axial, coronal or sagittal). The poor inter-observer agreement regarding the selected slice for the 2D measurement was illustrative for the complexity of performing uniform measurements in 2D. Especially patients, who sustained comminuted fractures (≥3 fragments), demonstrated a high dispersion of maximum gap and step-off measurements (Figs 6 and 7; patients 2, 5 and 10). Observers not only measured the gap and step-off at different 2DCT-slices, but also between different fragments. Q3DCT showed a lower dispersion in these patients in comparison with 2DCT. Except for patient 2, who had a small, severely displaced fragment (Fig. 1; purple fragment) that was disregarded by the observers in the 2D, but not in the 3D measurements. In contrast to 2D measurements, 3D analysis enables a complete and wider assessment of the fracture. Also, Q3DCT has the potential to provide improved quantitative information about the extent of the fracture.

The first parameter, the gap area (Fig. 4), is a reliable tool to quantify the total gap area of the fracture. In daily practice, the maximum fracture gap (mm) is mostly determined from a single CT slice in the coronal, sagittal, or axial direction. This 2DCT measurement is neither standardized, nor suitable for measuring multiple gaps, and therefore does not represent the entire injury. A discrepancy in the 3D gap area and 2DCT maximum gap due to multiple gaps in different directions is, for instance, demonstrated in patients 7 and 10 (Table 1, Fig. 3). The potential advantages of gap measurements from 3D are that they represent the gap of the entire fracture and can be used as a standardized quantitative measure of the extent of the fracture.

The second parameter, the articular surface involvement, was found to be a reliable parameter to assess how much of the articular surface was affected by the fracture. It is no surprise that as the Schatzker classification increased, the percentage of articular surface involvement gradually increased as well. However, substantial differences in articular surface involvement could be observed between patients who had similar fracture types. For instance, the articular surface involvement in all the cases with a Schatzker 2 injury varied from 12.7 to 39.7%. Therefore, this parameter could be a valuable addition to the currently used classification systems and might be of interest in fracture analysis within different subsets of fracture patterns.

The third parameter, 3D displacement, quantifies the displacement of each fracture fragment in all directions. The rationale behind exploring this parameter was that gaps and step-offs might arise from the displacement of these fragments. In this study, we demonstrated that it is possible to visualize the movement, tilting and rotation of each fracture fragment in three-dimensional space. For instance, this parameter might be of interest for research purposes regarding 3D analysis of specific parts of the fracture. The value of the total 3D displacement of all fracture fragments together, as a quantitative measure of the severity of the fracture, needs further exploration.

The Q3DCT method also has certain limitations. One of these is that the edges of the articular surface and the fracture lines still need to be determined manually in the current software program. An automatic edge detection tool for identifying the different fracture lines would be helpful to make these 3D measurements more consistent. A software package with automatic detection tools, snap to fit reduction tools and incorporated 3D measurements is probably the next step to avoid inter-observer variability in 3D fracture analysis. Of note is that the current workflow only utilizes a CT-scan of the affected knee. However, if a 3D model of the non-affected knee had been available as well, this could have been used as a reference when analyzing the injured side. Finally, the current process of creating the 3D model and performing the measurements takes at least half an hour, depending on the complexity of the fracture. However, further automation is being conducted for universal clinical applicability.

**Figure 8:**
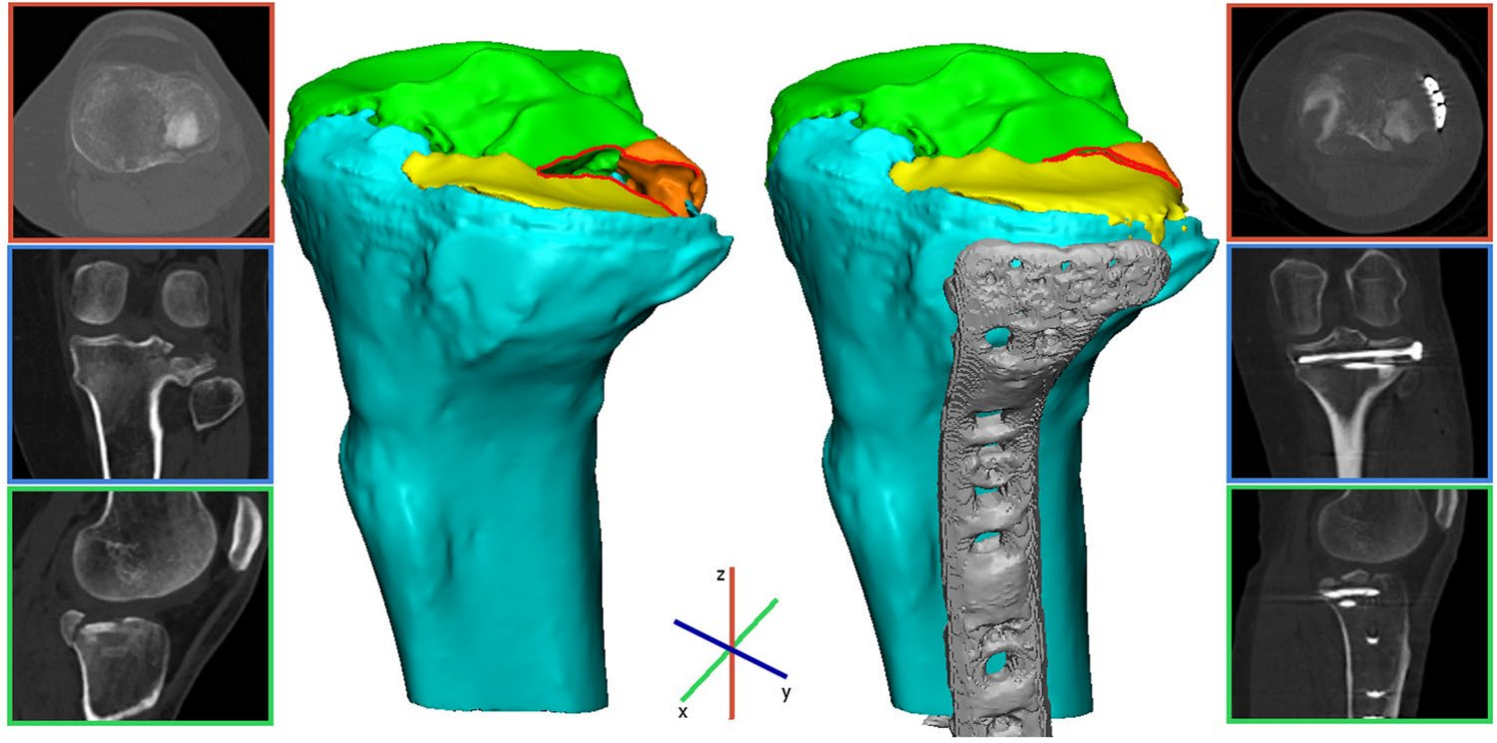
These images represent a clinical case in which we demonstrate the application of the quantitative 3D fracture measurements. Pre- and postoperative 3D assessment of a patient who was operated on a Schatzker 3 tibial plateau fracture. A 3D model of the pre-operative CT scan displayed a mean 3D gap of 0.5 mm, a step-off of 3.3 mm, a gap area of 27 mm^2^ with an articular surface involvement of 32.5% consisting of 3 fracture fragments. An open reduction and plate osteosynthesis was performed. A 3D model of the postoperative CT scan demonstrated an anatomical reduction of the fracture with a 20% decrease in the mean 3D gap (0.5 mm pre vs 0.4 mm postoperative), an 85% decrease in the mean 3D step-off (3.3 mm pre vs 0.5 mm postoperative), and a 10% decrease in the gap area (27 mm^2^ pre vs 24 mm^2^ postoperative).

Q3DCT measurements of the fractured tibial plateau could be considered an additional tool for the surgeon to assess and quantify preoperatively the extent of the fracture. The aim of this study was to present a standardized 3D measurement technique for tibial plateau fractures, which could be beneficial for further applications. These 3D measurements might also be helpful in comparing pre- and postoperative CT scans (if specifically requested) in order to assess the quality of the postoperative reduction. Finally, we included a case example where we applied our 3D measurements to pre- and postoperative CTs of a patient who was operated on a tibial plateau fracture in order to demonstrate that these can be used as a standardized quantitative measure for assessing the quality of the postoperative reduction (Fig. 8). Furthermore, the connection between quantitative 3D measurements and patient reported outcomes might be of interest to surgeons to reassess fracture parameters and then decide whether to proceed to an operative treatment regimen.

## Conclusion

We present a feasible and reliable method for Q3DCT fracture analysis of tibial plateau fractures. These 3D measurements can, potentially, be used to assess complex multi-directional injuries and to quantify the extent of the fracture.

## Data Availability

The authors declare that the data supporting the findings of this study are available within the paper.
